# SiC Measurements of Electron Energy by *fs* Laser Irradiation of Thin Foils

**DOI:** 10.3390/mi14040811

**Published:** 2023-04-02

**Authors:** Lorenzo Torrisi, Mariapompea Cutroneo, Alfio Torrisi

**Affiliations:** 1 Department of Mathematics and Computer Sciences, Physical Sciences and Earth Sciences (MIFT), University of Messina, V.le F.S. D’Alcontres 31, 98166 Messina, Italy; lorenzo.torrisi@unime.it; 2 Nuclear Physics Institute of the CAS, Hlavní 130, 250 68 Husinec-Řež, Czech Republic; cutroneo@ujf.cas.cz; 3 Dipartimento Interateneo di Fisica, Università di Bari “Aldo Moro”, 70125 Bari, Italy; 4 Istituto Nazionale di Fisica Nucleare (INFN), Sezione di Bari, 70126 Bari, Italy

**Keywords:** electron acceleration, electron detection, graphene oxide, laser-generated plasma, Schottky diode, silicon carbide, TNSA

## Abstract

SiC detectors based on a Schottky junction represent useful devices to characterize fast laser-generated plasmas. High-intensity *fs* lasers have been used to irradiate thin foils and to characterize the produced accelerated electrons and ions in the target normal sheath acceleration (TNSA) regime, detecting their emission in the forward direction and at different angles with respect to the normal to the target surface. The electrons’ energies have been measured using relativistic relationships applied to their velocity measured by SiC detectors in the time-of-flight (TOF) approach. In view of their high energy resolution, high energy gap, low leakage current, and high response velocity, SiC detectors reveal UV and X-rays, electrons, and ions emitted from the generated laser plasma. The electron and ion emissions can be characterized by energy through the measure of the particle velocities with a limitation at electron relativistic energies since they proceed at a velocity near that of the speed of light and overlap the plasma photon detection. The crucial discrimination between electrons and protons, which are the fastest ions emitted from the plasma, can be well resolved using SiC diodes. Such detectors enable the monitoring of the high ion acceleration obtained using high laser contrast and the absence of ion acceleration using low laser contrast, as presented and discussed.

## 1. Introduction

*fs* laser-generated plasmas have been largely studied in the last ten years, with special regard to the production of accelerated protons, electrons, and X-rays [1]. In order to obtain high proton acceleration, useful for many applications (radiotherapy, surface beam analysis, nuclear reactions and nuclear fusion processes, etc.), the target normal sheath acceleration (TNSA) regime has been promoted by irradiating thin foils and generating photons and high energy particle emission in the forward direction (rear side of the target) [2,3,4,5]. The employment of hydrogenated thin targets to produce high proton energies and currents is particularly interesting, reaching actual values in the order of 100 MeV and mA, respectively [6]. Moreover, the high X-ray intensity, produced in a very short time, constitutes a singular non-monochromatic source of X-rays, which can be used for radiotherapy treatments to facilitate fast radiographic exposures and assist the analysis of different types of materials [7]. In terms of the energy distribution, flux, and angular distribution of emitted X-rays, electrons, and ions, the plasma characteristics depend strongly on the laser parameters, irradiating conditions, and target properties. By varying these parameters, it is feasible to change the temperature and density of the plasma vs. time and distance from the target, the electric field driving the ion acceleration, the ion charge state distribution, the particle angular emission, and other quantities [8].

The fast-generated plasmas can be characterized using several techniques, such as CCD visible and X-ray streak cameras, Thomson parabola spectrometers [9], Ion energy analyzed, Faraday cups, Gafchromic films and track detectors, optical spectroscopies, interferometric imaging, and different types of semiconductor detectors [10,11]. Particularly, the SiC semiconductor is gathering wide interest for the monitoring of short plasma duration, having high temperature and density, and emitting energetic and intense spectra of UV and X-rays, electrons, and low and high energy ions [12,13]. The employment of SiC detectors for plasma diagnostics is very attractive because they offer the advantage of not being sensitive to the high emission of visible, soft ultraviolet (UV), and infrared light plasma emitted. In fact, such photons are not able to produce electron–hole pairs because their energies are below the 3.3 eV value of the 4H–SiC gap energy [14].

Moreover, SiC detectors offer the advantages of operating under high temperatures, being highly resistant to defect generation, having a low reverse current, and operating with a high energy resolution, as reported in the literature [15]. By using a time-of-flight (TOF) detection configuration, the SiC diodes enable the measurements of the velocity of the particles emitted from the plasma source, and they are being used for measurements in plasmas generated by high-intensity lasers irradiating solid targets [16].

The detection of electron emission from laser-generated plasma represents a not simple aspect due to the high electron velocity, their very low time duration and high intensity, and their relativistic effects when produced by laser intensities above 10^15^ W/cm^2^. They can be detected using a Thomson parabola spectrometer, fast scintillators, X-ray CCD streak cameras, Gafchromic films, optical spectrometers, interference imaging, and Faraday cups [17,18]. The measure of their energy is tricky because their response is often superimposed on light, UV, X-ray, and ion detection and often involves wide energy ranges. The electromagnetic wave of the laser light pushes through and separates the electrons from the plasma, generating coherent relativistic electron emission. The hot electron cloud and the ions in the target generate an intense electric field that drives the ion acceleration towards the electron cloud. The electrons acquire speed and, from their initial positions at rest, accelerate away from the target at an astonishing speed close to the speed of light. This acceleration allows very high energies to be reached over very small distances, in the order of millimeters. The electron laser wakefield acceleration (LWFA) in plasmas is obtaining the highest energy gains, reaching teens of GeVs. The use of short laser pulses and repetitive pulses support the generation of electron beams nearly monochromatic and high current beneficial for many applications such as light sources, high-energy physics, radiotherapy, 3D imaging, materials treatment and analysis, and others [19].

In this paper, some TOF spectra obtained using SiC Schottky diodes to detect photons and electrons emitted from *fs* lasers generating plasma in a high vacuum are reported. The electron energy and energy interval are measured using a relativistic approach. The presented data are intended to prove the high sensitivity of SiC to the fast and slow electron emission produced by sub-ns laser-generated plasma in the TNSA regime.

## 2. Materials and Methods

SiC detectors have been used as Schottky diodes, built on 4H–SiC n-type epitaxial layers with very low defects concentration and a carrier concentration of about 10^14^ cm^−3^, a thickness of 80 μm, and grown on a highly doped n-type SiC substrate, as similar detectors presented in the literature [20]. The backside ohmic contact uses a 100 nm thick Ni film. The frontside contact, realized using standard photolithography, uses a 200 nm nickel silicide layer (Ni_2_Si) to optimize the Schottky barrier properties. The SiC Schottky diodes were characterized by current–voltage (I–V) characteristics, giving a barrier height of 1.65 eV and a low reverse current density of about 10 nA/cm^2^ at 700 V reverse bias and 22 °C working temperature.

**Figure 1:**
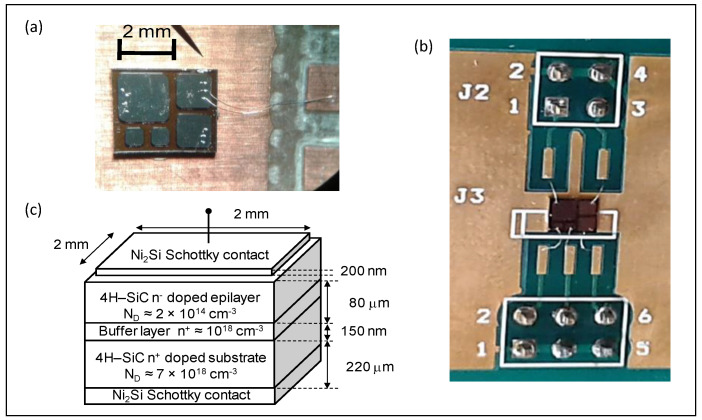
Photo of the SiC detector employed, the larger one of the five reported (**a**); holder of the SiC detectors (**b**); and scheme of the SiC diode employed in this experiment (**c**).

**Figure 2:**
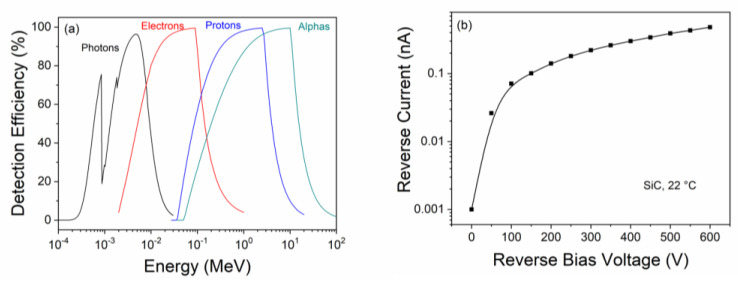
SiC detection efficiency versus energy for different incident radiations (**a**) and the reverse current versus the reverse bias voltage at room temperature (**b**).

In our experiment, the active area of the SiC detector was 2 mm × 2 mm, and the used reverse bias was 600 V, which produces a depletion layer of about 55 μm. In such a configuration, the detector has a high energy resolution, with a full-width-half-maximum (FWHM) of 34 eV, high linearity with the radiation energy, and 100% charge collection efficiency, as demonstrated by detecting alpha particles emitted from radioactive sources [21]. A photo of the single device (the larger one with respect to the other four) and of all device holders is shown in Figure 1a,b, respectively. Figure 1c shows the scheme of the SiC Schottky diode employed in these measurements, as already reported in [16]. Generally, its reverse bias was maintained at −600 V. Figure 2 shows the detection efficiency of the SiC detector as a function of the energy of X-rays, electrons, protons, and helium beams (a) [16], and the reverse current versus the reverse bias voltage at room temperature (22 °C) (b). The detection efficiency was calculated as a function of the energy evaluating the energy release of photons, electrons, protons, and alpha particles in the SiC active thickness of 80 μm. For this calculation, CXRO [22] and NIST databases [23], SREM [24], and SRIM [25] codes have been used. It is possible to observe that at the used experimental conditions, the detection efficiency is high for photons up to about 20 keV, electrons up to about 1 MeV, and protons up to about 10 MeV.

The SiC detector was employed in the TOF regime using as a start the laser pulse and as a stop the particle detection peak. To this, the flight distance target–detector was fixed, generally at d = 82 cm, and a fast storage oscilloscope was employed (Tektronix, 20 GS/s, 100 MHz). At this distance, the solid angle submitted by the SiC detector is 6.25 μSr. SiC was coupled to the 50 Ω oscilloscope input through 1 nF capacitance, as reported in [26]. Its temporal resolution is better than 1 ns. The incident laser beam is 0° (really 1° to avoid reflections toward the laser system) while two SiC detectors were employed to monitor the TOF plasma emissions: SiC 1 in the backward direction at 82 cm distance from the target and at an angle generally of −25°; SiC 2 in the forward direction at 82 cm distance from the target and at angles between 0° and +25°.

**Figure 3:**
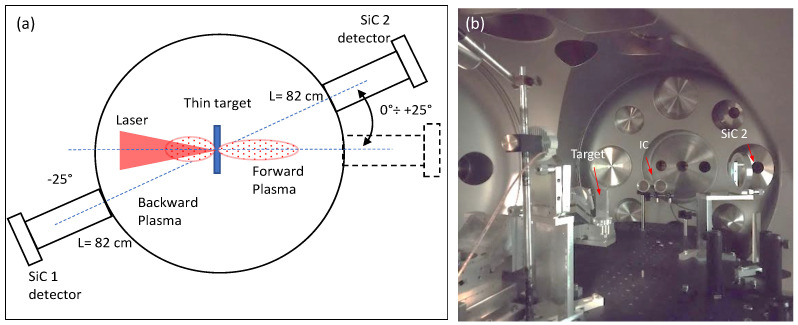
Scheme of the experimental setup (**a**) and a photo of the scattering chamber and of the thin target in the holder at IPPLM laboratory (**b**).

Experiments were performed employing a Ti-sapphire laser operating at 800 nm fundamental wavelength, with 40 fs pulse duration (FWHM), in a single pulse, and at a laser pulse energy ranging between 100 mJ and 300 mJ. The laser focal spot was 10 μm in diameter, and the laser beam focal distance (FP) from the target surface generally was maintained at 0 microns, but also other values have been tested. The maximum laser intensity corresponds to about 6.4 × 10^18^ W/cm^2^. In the first laboratory, the CELIA of Bordeaux (France) [27], the laser contrast was very low and of about 10^−5^, while in the second laboratory, the IPPLM of Warsaw (Poland) [28], it was high and of about 10^−9^. In the first laboratory, the high pedestal produces high electron emission at low energy, which is responsible for the not ion acceleration, while in the second laboratory, the pedestal is negligible with respect to the main *fs* laser peak, and the fast electron emission produces high ion acceleration along the normal to the target surface [19,29]. A scheme of the experimental set-up, with forward collection radiation and angles of 0° ÷ +25°, backward detection at −25°, and flight length of 82 cm, is shown in Figure 3a, while Figure 3b shows a photo of the scattering chamber and of the thin target in the holder at IPPLM laboratory. Two similar SiC detectors have been employed, the first in the backward direction, SiC 1, and the second in the forward one, SiC 2, with the same characteristics.

The thin targets to be laser irradiated were prepared with an advanced technology employing nanoparticles (NPs) at the MIFT Department of Messina University. They consist of hydrogenated thin polymeric foils based on graphene oxide (GO) foils covered with Au thin films [30]. GO is rich in carbon, oxygen, and hydrogen elements. It was 7 μm thick and was covered in both faces with 100 nm Au thin films in order to enhance the target electron density. In fact, as reported in the literature, the electric field *E* driving the forward ion acceleration developed in front of the target surface depends on the equation [8]:

(1) $$E=\sqrt{\frac{n_{e}kT}{\varepsilon_{0}}}$$

where *n_e_* is the plasma electron density, *ε*_0_ is the vacuum permittivity and *kT* is the equivalent plasma temperature.

Another employed thin target was polyethylene (PE, (CH_2_)_n_) based. To accelerate high-energy protons from this polymer, heavy metallic nanoparticles have been embedded into PE [31] to increase its electron density. To this, 10 nm diameter spherical gold nanoparticles at a concentration of about 1 wt % were employed. The target thickness has been 6 μm, at which the proton emission has shown high energy acceleration values above 1 MeV. The foil thickness was chosen on the basis of our previous measurements at IPPLM, which allowed us to optimize such thickness to obtain a high proton acceleration in the TNSA regime. [16,32]. The electrons’ energies have been evaluated by measuring their velocities from the TOF measurements (*v* = d/Δt_TOF_) and calculating their kinetic energies *E* by the relativistic formulation:

(2) $$E=\frac{1}{2}\;\frac{m_{e}}{\sqrt{1-{\left(\frac{v}{c}\right)}^{2}}}v^{2}.$$

Here, *m_e_* is the electron mass, *v* is the measured electron velocity, and *c* is the speed of light.

## 3. Results and Discussion

**Figure 4:**
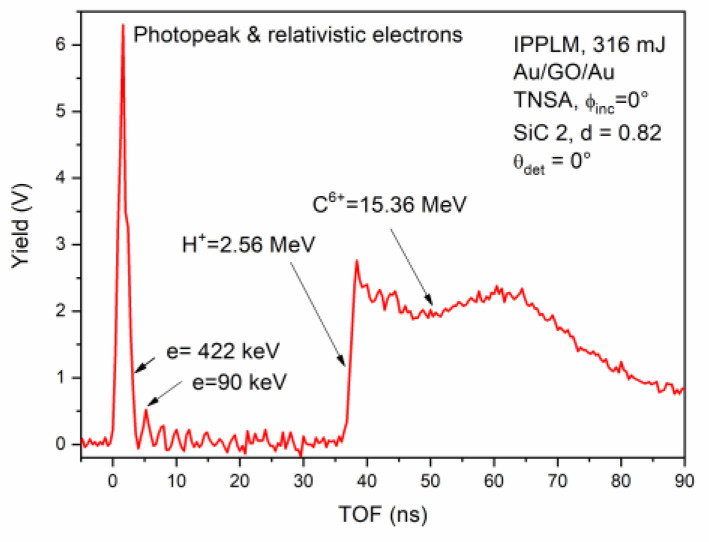
SiC-TOF forward spectrum obtained by irradiating a thin foil of Au/GO/Au 7 μm thick in the TNSA regime at high laser contrast.

A typical SiC-TOF spectrum acquired in the IPPLM laboratory detecting plasma emission in the forward direction using SiC 2 and 316 mJ laser pulse energy, irradiating an advanced target constituted by a graphene oxide (GO) foil, 7 μm thick, covered in both faces with a thin Au foil, 200 nm thick, is displayed in Figure 4.

The incidence of the laser beam is 0° while the detection is at 0° in the forward, i.e., along the normal to the target surface, where the ion acceleration has maximum energy and yield. The spectrum shows a main narrow peak (photopeak) due to the laser light, X-rays plasma produced, and relativistic accelerated electrons, which is used as a trigger signal for the storage oscilloscope of the plasma emission detection. The detected electrons have energy (calculated using Equation (2)) higher than about 400 keV; however, slow electrons at about 90 keV and less are also disclosed. The target’s emitted electron cloud is very energetic, nearly monochromatic, and leads the ion emission from the target, which is expected at high energy. This peak, in fact, is followed by a negligible background due to less energetic electron detection and by a large and intense peak due to ion detection. The faster ions are protons due to the high hydrogen concentration present in the GO target, and the slower ions are due to the accelerated carbon ions from C^6+^, the faster, up to C^1+^, and the slower, coming from the graphene target, as reported in our previous papers [16].

In previous papers, using a Thomson parabola spectrometer, the authors have demonstrated that the TNSA forward carbon ion acceleration from C^1+^ up to C^6+^ occurs [33]. By considering the measured TOF and the flight distance of 82 cm, it is possible to evaluate the presence of electrons from more than 1 MeV, up to about 100 keV or less, and a maximum proton energy, measurable from the position of the ion peak growth, at about 37 ns, corresponding to 2.56 MeV. The ion peak shows a convolution of protons and carbon ions, indicating that the faster carbon ions have a maximum energy of about 15.36 MeV. This last evaluation confirms that the faster C ions have an energy corresponding to their charge state 6+ by the ion acceleration of 2.56 MeV per charge state measured for protons, in agreement with the literature [16]. The long tail of the carbon peak is due to the SiC detection of the six charge states of carbon ions with energies proportional to their charge state, up to C^+^ ions. Each ion has a large ion energy distribution like a Boltzmann–Coulomb-shifted (CBS) one described in the literature [32].

For good ion acceleration, such as that presented in Figure 4, the reproducibility of the experiment is high and evaluated in the order of 90%. However, in general, it is lower due to the laser instability, changes in laser focalization with respect to the target surface, and target non-homogeneity in composition and thickness.

**Figure 5:**
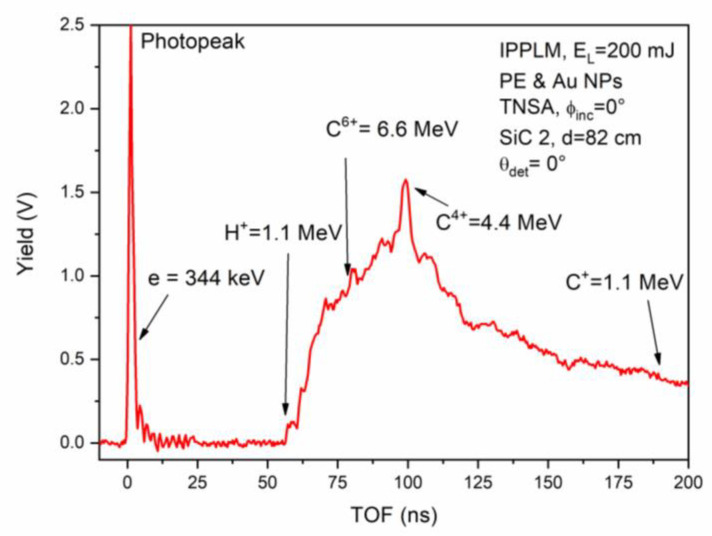
SiC-TOF forward spectrum obtained by irradiating a thin foil of PE+ Au NPs 6 μm thick in the TNSA regime at high laser contrast.

Another analysis performed at the IPPLM laser laboratory concerns the PE irradiation in the TNSA regime using the same experimental conditions. Additionally, in this case, thanks to the high laser contrast, the photopeak is very narrow, demonstrating very good ion acceleration in the normal direction, as shown in Figure 5.

The forward spectrum shows a photopeak due to the detection of X-rays and fast electrons, which last with energies within about 1 MeV and 344 keV. The ion peak evinces fast protons at a maximum energy of about 1.1 MeV and fast carbon ions with a maximum energy of about 6.6 MeV, corresponding to the acceleration of C^6+^ ions. Thus, as expected, the proton energy is lower with respect to the previous spectrum shown in Figure 4 because the ion acceleration, depending on the driving of the developed electric field pulse (calculable from Equation (1)), increases with the plasma (i.e., target) electronic density, which is lower in the case of the polyethylene foil. The maximum carbon ion kinetic energies enhance with their charge state with the factor 1.1 MeV per charge state. The reported data are consistent with the literature [32].

To better evince the electron emission from the target and the high-efficiency response of SiC to the electron detection, some experiments have been performed in the CELIA laser laboratory at low laser contrast producing electron acceleration both by the pedestal laser pulse and by the main *fs* laser pulse [34]. In this case, the electron emission is characterized by a lower energy, a larger energy distribution, and a larger angular distribution compared with the high contrast lasers, which generally do not assist high ion acceleration. The laser incidence was maintained at 0°, and the detector was placed at different angles within ±25°, as reported in Figure 2a.

**Figure 6:**
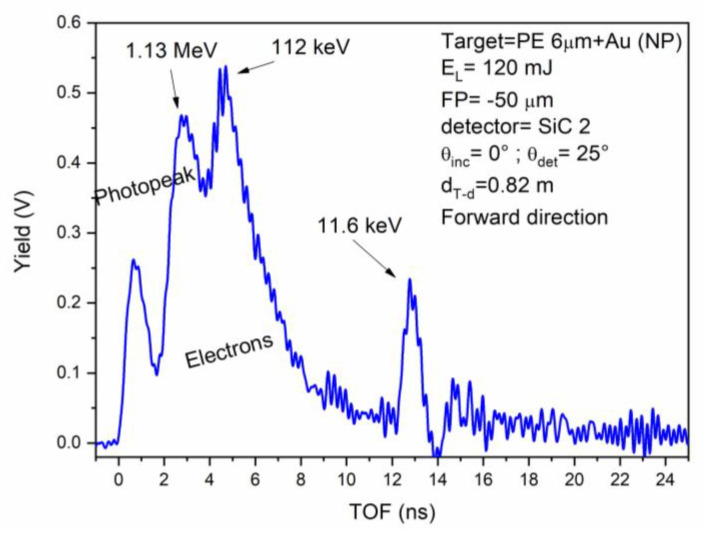
SiC-TOF forward spectrum obtained by irradiating a thin foil of PE with Au NPs, 6 μm thick in the TNSA regime, and low laser contrast.

Figure 6 shows a forward SiC spectrum obtained using the low-contrast laser irradiating the above-described target (PE + Au NPs, 6 μm thick). The laser pulse energy was E_L_ = 120 mJ, the incidence laser angle 0°, the detection angle 0°, and the flight distance of 82 cm. In this case, the photopeak is well separated by the multiple electron peaks, which detect hot and cold electrons. The hot electrons at 1.13 MeV energy are those accelerated by the main laser pulse, while the successive peak centered to about 112 keV showing the presence of cold electrons could be due to the laser prepulse acceleration. The less energetic peak centered at about 11.6 keV probably is due to the preplasma electron emission or to electron reflection and scattering effects from the stainless-steel walls of the large vacuum chamber. The faster peak is centered at 2.8 ns with a width (FWHM) of 2 ns, the second is centered at 4.6 ns with a width of 2.5 ns, and the third at 12.9 ns with a width of 1 ns. The mean energy of the three electron peaks has been calculated using Equation (2). This result is interesting because it indicates that the main peak accelerates electrons to a kinetic energy of about 1 MeV, but other less energetic electrons are generated by the pulse pedestal and by the induced preplasma, disturbing the ion acceleration process. In fact, no protons are present in the spectrum either if the detection angle is changed between 0° and 26° from the normal to the target surface and the focal position is modified from −100 μm (in front of the target) up to +100 μm (inside the target).

**Figure 7:**
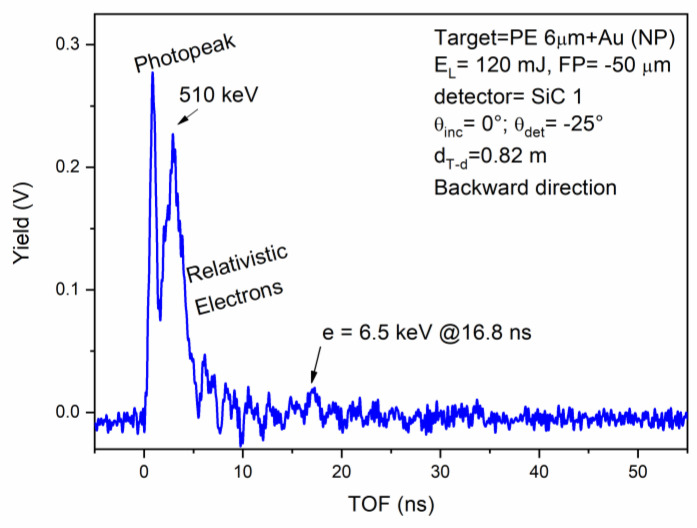
SiC-TOF backward spectrum obtained by irradiating a thin foil of PE with Au NPs, 6 μm thick in TNSA regime, and low laser contrast.

The electron emission by low laser contrast has also been measured in the backward direction, noting that even in this direction, their energy remains low. Figure 7 shows the backward SiC spectrum obtained irradiating the above-described target (PE + Au NPs, 6 μm thick) in the previous laser conditions, i.e., low laser contrast (10^−5^), laser energy pulse E_L_ = 120 mJ, SiC 1 detector position −25°, and flight distance d = 82 cm.

In this case, the photopeak is time separated by the fast-accelerated electron peak, being this last centered at about 3 ns with a width of about 2.5 ns. It means that the electron kinetic energy is centered at about 510 keV, indicating that the backward electron energy is lower than the forward one, according to the literature data [35]. The presence of reflected electrons is absent, probably due to the larger vacuum chamber in this direction. However, a small peak at about 16.8 ns indicates the presence of electrons bunching at about 6.5 keV, which may have been reflected at low energy and yield. No ion acceleration is detected at higher TOF values.

At low laser contrast, different tests have been performed to try to accelerate protons using the two types of targets presented above, but none of them gave positive results. In fact, the SiC detector has always confirmed the production of electrons emitted at large angles around the normal direction and not well monochromatic, as a result of the high preplasma emission from the laser pedestal justifying the not detected forward and backward ion acceleration.

**Figure 8:**
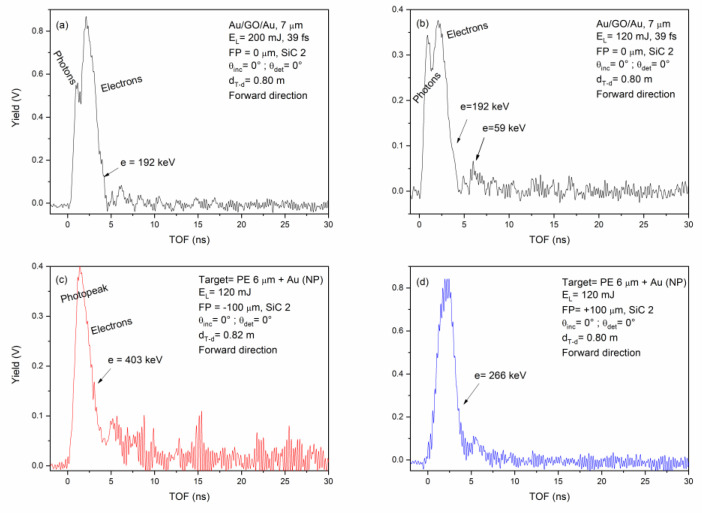
SiC-TOF spectra in the forward direction and 0° detection angle by irradiating Au/GO/Au foil at 120 mJ (**a**) and 200 mJ (**b**) with FP = 0 μm and irradiating Au/GO/Au foil at 120 mJ with FP = −100 μm (**c**) and FP = +100 μm (**d**).

Figure 8 reports two SiC spectra obtained irradiating the Au/GO/Au 7 μm thick target at low laser contrast, at 0° incidence angle, 0° forward detection angle, using a pulse energy of 120 mJ (a) and 200 mJ (b), and FP = 0 μm (focus on the target surface). Irradiations at different laser focal positions with respect to the target surface have been performed, but again proton acceleration was not obtained. The electron peak in both cases is separated from the photopeak, demonstrating an energy lower than 1 MeV and insufficient to induce the high electric field needed for the forward ion acceleration, according to the literature [12].

Figure 8 also shows the SiC-TOF spectra coming from the laser irradiation of the PE + AuNPs 6 μm foil at 120 mJ pulse energy with FP = −100 μm (c) in front of the target surface and FP = +100 μm (d) inside the target surface, respectively. In such cases, the photopeak is near the electron peak, and their convolution is detected as a large initial peak. From this, an evaluation of the minimum electron energy up to about 190 keV can be performed. Such a measure indicates that electron energy is too low for the development of the high electric field needed to produce high ion acceleration in the forward direction.

In these reported cases, the photopeak is always accomplished by electrons with energy in the order of hundred keVs, too slow and not sufficiently monochromatic as required to generate an efficient electric field pulse able to accelerate the emitted target ions. The electron detection evidences low energy electrons produced at large angles from the laser pedestal, which cannot achieve an electric field impulse high enough to accelerate ions.

This electric field, in fact, is too spatially developed (for non-monochromatic electrons) and too temporally extended (tens of ns) to drive a significant ion acceleration. 

**Figure 9:**
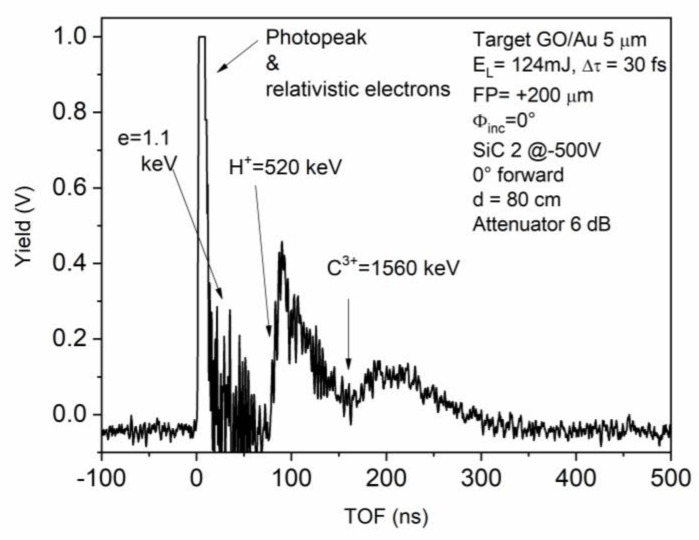
SiC-TOF obtained by irradiating a GO/Au thin foil in the TNSA regime at Celia Laboratory.

Thus, using low laser contrast, generally, no ion acceleration or low ion acceleration occurs. However, by reducing the pedestal duration and prepulse intensity and increasing the main laser pulse energy, it is possible to obtain a significant proton acceleration, as reported in the literature [36,37,38]. Figure 9 shows a SiC-TOF TNSA forward spectrum obtained at the CELIA laboratory using a short and less intense laser pedestal, increasing the laser contrast to about 10^−6^ and reducing the preplasma formation, and irradiating an advanced target constituted by 1 μm graphene oxide (GO) covering 5 μm Au thin foil using a laser pulse of 124 mJ and a main pulse width of 30 fs.

In such conditions, the SiC detector can detect the photopeak and the relativistic electrons and allows measurement of the maximum proton and carbon ion energies in the TOF spectrum, corresponding to 520 keV and 1560 keV, respectively, identifying a plasma acceleration of 520 keV per charge state.

## 4. Conclusions

The presented results reveal two important aspects, one related to the physics of the plasma produced by high-intensity *fs* lasers irradiating thin advanced foils in TNSA conditions, and the other validates the use of SiC detectors to characterize the produced plasma pulse. In particular, this second aspect is more important to clarify in the TOF approach, SiC can detect the electron emission from the laser–matter interaction and recognize if the electric field driving the ion acceleration is developed or not. The formation of a narrow photopeak containing X-rays and relativistic electrons, with energy higher than 1 MeV, indicates that the forward ion acceleration may occur, as demonstrated by the proton and light ion acceleration at IPPLM laboratory using a high-contrast *fs* laser. The formation of a narrow photopeak containing X-rays and relativistic electrons with energy higher than 1 MeV supports, in fact, the acceleration of good proton and other light ions, as observed by the high laser contrast at IPPLM laboratory. On the contrary, the formation of a large photopeak containing electrons at energies in the order of hundred keVs, generated by the pedestal of the low contrast laser, does not allow one to obtain an electric field pulse so high as to accelerate ions because it is too spatially and temporally distributed.

As a consequence of the high energy gap of silicon carbide, which does not allow the detection of the high intensity of visible light emitted by the plasma, which gives a low reverse current also at relatively high temperatures, the detector is very sensitive to the radiations that invest it, even at low fluences. Moreover, SiC shows high speed of response and high temporal resolution due to the rapid collection of created pairs. Finally, SiC provides deeper insight in the mechanisms of plasma formation and particle acceleration which occur in very short times, especially by using *fs* lasers irradiating thin foils.

## Data Availability

Not applicable.
